# Fluid balance in pediatric critically ill patients (with and without kidney dysfunction)

**DOI:** 10.1097/MCC.0000000000000987

**Published:** 2022-09-27

**Authors:** Zaccaria Ricci, Erica Bjornstad

**Affiliations:** a Pediatric Intensive Care Unit, Meyer Children's University Hospital; b Department of Health Sciences, Section of Anesthesiology and Intensive Care, University of Florence, Florence, Italy; c Department of Pediatrics, Division of Nephrology, University of Alabama at Birmingham, Birmingham, Alabama, USA

**Keywords:** acute kidney injury, acute respiratory distress syndrome, extracorporeal membrane oxygenation, fluid balance, fluid overload

## Abstract

**Purpose of review:**

The issues of fluid balance and fluid overload are currently considered crucial aspects of pediatric critically ill patients’ care.

**Recent findings:**

This review describes current understanding of fluid management in critically ill children in terms of fluid balance and fluid overload and its effects on patients’ outcomes. The review describes current evidence surrounding definitions, monitoring, and treatment of positive fluid balance. In particular, the review focuses on specific patient conditions, including perioperative cardiac surgery, severe acute respiratory failure, and extracorporeal membrane oxygenation therapy, as the ones at highest risk of developing fluid overload and poor clinical outcomes. Gaps in understanding include specific thresholds at which fluid overload occurs in all critically ill children or specific populations and optimal timing of decongestion of positive fluid balance.

**Summary:**

Current evidence on fluid balance in critically ill children is mainly based on retrospective and observational studies, and intense research should be recommended in this important field. In theory, active decongestion of patients with fluid overload could improve mortality and other clinical outcomes, but randomized trials or advanced pragmatic studies are needed to better understand the optimal timing, patient characteristics, and tools to achieve this.

## INTRODUCTION

Fluid homeostasis is critical in the care of severely ill children. Too much or too little fluid was recognized as far back as Hippocrates as important to a patient's health; balance was the key. To this day, we continue to learn more about the pathophysiology of abnormal fluid balance, optimal management, and interaction with kidney dysfunction and patient outcomes. Over the past 2 years, there has been an exponential emphasis on understanding fluid status and its interaction with acute kidney injury (AKI) and patient-related outcomes; since 2020 over 500 articles have been published on fluid status and AKI, which makes up more than one-third of the articles indexed on the topic on PubMed.

Fluid balance (FB) and AKI are a balancing act. Dehydration, or a detrimental negative fluid balance, can lead to AKI, whereas AKI itself can lead to and risk fluid overload, or a positive fluid balance with negative patient implications. AKI in and of itself does not cause fluid overload but the combination of AKI with iatrogenic administration of excessive fluids can cause fluid overload. A positive FB with a normal functioning kidney will (with time) correct itself to euvolemia, but if this positive FB occurs with any kidney dysfunction, then, if left unchecked, it will ultimately result in unfavorable clinical outcomes. This has been at the center of recent observational cohort studies demonstrating worse outcomes among critically ill patients with fluid overload [1]. 

**Box 1:**
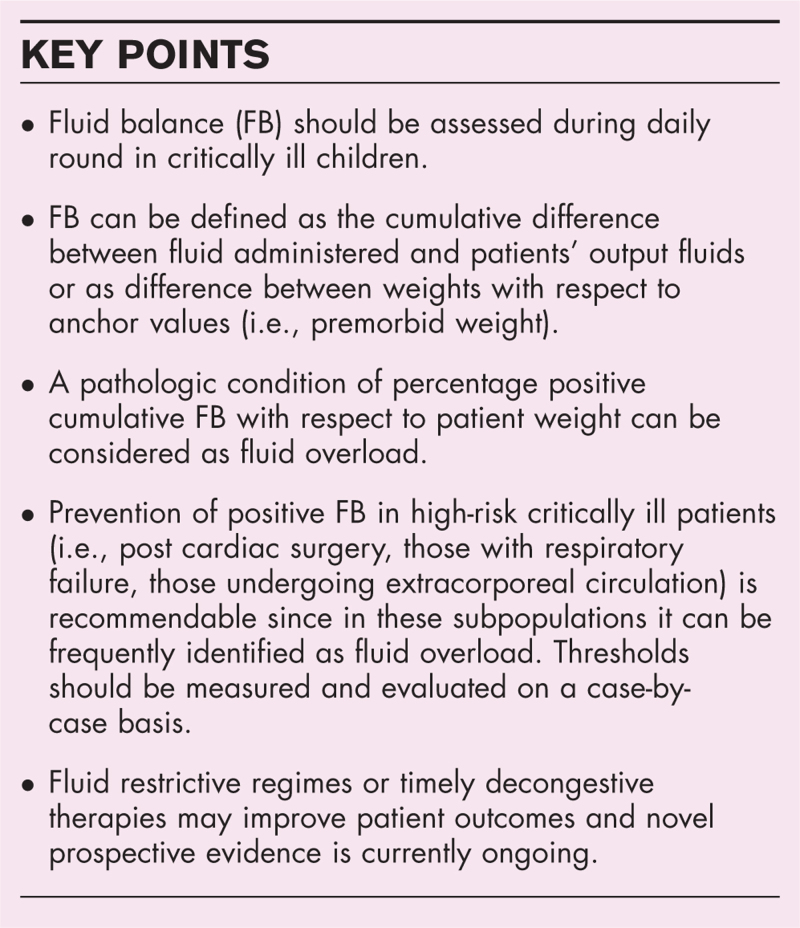
no caption available

## FLUID BALANCE AND FLUID OVERLOAD: DEFINITION AND MEASUREMENT

Positive FB (or fluid accumulation) alone is present when the difference between fluids in and fluids out is positive. This condition should be clearly distinguished from a positive FB with detrimental clinical consequences, that is, fluid overload. For example, a sick child who presents severely dehydrated with weight of 12 kg, may then receive over 1.5 l of fluids in the first 24 h with a weight gain of 1 kg. However, if the child's weight was 14 kg just 2 months ago at a well child visit and this was due to acute gastroenteritis, the definition of fluid overload at this point might not hold based on our common measurement methods (weight-based or metric-based) and the patients might reasonably be considered as a positive FB. There is not yet a standard approach to defining FB or fluid overload and from the nine pediatric-focused original research articles published from 2020 through March 2022 there were more than 12 different definitions with multiple additional combinations possible [2^▪^,^3^,4^▪^,^5^^▪▪^,^6^,^7^^▪▪^,^8^,9^▪^,10^▪^] (Table 1). The different definitions included metric vs. weight-based methods, considered different baseline (or anchor) weight, and different percentage thresholds (e.g., 10% vs. 20% vs. continuous). The exact day from pediatric intensive care unit (PICU) admission when the measurement should be performed is also a matter of uncertainty (e.g., hospital day 3 or 7) (Table 1). Finally, only half of the metric-based methods exactly delineated the fluids that counted for inputs and outputs, and only a quarter of them described how urine output was captured (i.e., whether or not an invasive Foley catheter was used and if not, how volume was calculated consistently across patients).

**Table 1:** Summary of pediatric articles related to fluid balance (FB)/overload (FO) and acute kidney injury (AKI) by definitions used in publications

Author	Year	Type of study design, # of centers	Fluid balance measurement type (metric vs. weight-based)	Fluid balance/ overload definition	Clinical cut-offs	Specified types of fluid for metric measurements (i.e., includes stool?)	Specified if Foley catheters used for output measurements
Gist [2^▪^]	2020	Secondary analysis of prospective cohort, single center	Metric	% FO daily = (fluid in – fluid out)/PICU admission weight	FO ≥ 20%Day 3 for primary outcome	No	Specified in primary study: Foley in place for at least first 48 h
Rajapreyar [3]	2021	Retrospective cohort, multicenter	Not defined	Not defined	Not defined	No	Not specified
Stulce [4^▪^]	2020	Retrospective cohort, single center	Metric	FB = (fluid in – fluid out)/ admission weight (or estimated from ED)	FO ≥ 10% at day 10 or death	Yes	Not specified
Gorga [5^▪▪^]	2020	Retrospective cohort, multicenter	Metric	% FO = sum daily (fluid in – out)/PICU admission weightFor 28d before ECMO, 21-day post-cannulation	Cumulative FO at CRRT initiation, at CRRT discontinuous	No	Not specified
Zanaboni [6]	2021	Retrospective cohort, single	Metric	FB = (total in – out)/preop weight	Peak FO = cumulative FB on postop day 3Negative FB = first day FB is negative	No	Not specified
Barhight [7^▪▪^]	2022	Retrospective, two centers	Metric	% FO = (cumulative fluid in – out)/PICU admission weight	Nonresus vol in excess = ml in excess of maintenance (defined by 4–2–1)	Yes	Not specified
Flood [8]	2021	Retrospective, single center	Weight-based	Maximum cumulative % FO = (max weight – nadir weight)/nadir weight	FO ≥ 10%	n/a	n/a
Al-lawati [9^▪^]	2020	Prospective, single center	Metric	% FO = cumulative (fluid in – out)/PICU admission weight	FO ≥ 15%	Yes	Not specified
Gorga [10^▪^]	2021	Secondary analysis of prospective cohort	Metric	% FO = cumulative (fluid in – out)/PICU admission weight	FO ≥ 15% on day 3	Yes	Not all had a Foley, but clearly specified how they dealt with voids not captured and diapered children

CRRT, continuous renal replacement therapy; ECMO, extracorporeal membrane oxygenation; ED, emergency department; PICU, pediatric intensive care unit.

Common methods for defining total body fluid status are either weight-based (assessing changes in a patient's weight over a specified number of days) or metric-based (assessing mathematical calculations of total fluids a patient receives – input – and outputs), which may or may not be indexed to a baseline weight to give a percentage [11]. With these methods, recent literature is showing a consistent positive association between positive FB and adverse patient outcomes (mortality, length of stay, increasing ventilatory support, AKI) [12].

However, other studies have shown that this association does not always hold. Two recent U.S.-based retrospective studies have found that on single analysis, AKI and positive fluid balance are associated with adverse outcomes (prolonged ICU length of stay, mortality) but when controlling for additional confounders these associations are no longer significant [2^▪^,4^▪^]. A recent meta-analysis of neonates with abnormal fluid balance found that the associations depend on the sub-set of populations; there were higher associations of fluid overload and mortality in the congenital cardiac cohort and those at continuous renal replacement therapy (CRRT) initiation [13^▪^]. Both cohorts are unique and should not be generalized to all neonates as general term and preterm infants did not have an association between fluid overload and mortality. A retrospective study tried to evaluate interventions and found that pediatric patients with hemolytic uremic syndrome were less likely to require dialysis if they were in a volume expansion group compared to a fluid restricted group (absolute risk reduction of 0.34, 95% confidence interval (CI) 0.07–0.63, with associated number needed to treat of three patients with volume expansion to prevent one patient from needing dialysis) [14^▪▪^].

There are potential reasons for the contradictory findings in these observational studies. There is not a standard approach to measuring, defining and hence reporting fluid balance across pediatric cohorts, distinct cohorts may respond differently to different levels of abnormal fluid balance, and unmeasured or unrecognized confounding or other sources of bias may limit the findings of the studies. Therefore, these issues raise concern about translating observational studies directly into clinical care. Another potential reason is inherent in the assumption that total body FB is synonymous with intravascular FB. Therefore, we must be careful before clinically applying observational study data that only assesses total body fluid status. Randomized trials or other pragmatic rigorous evaluations are needed before widespread clinical adoption that all positive FB is bad.

The pediatric Pediatric Emergency Care Applied Research Network (PECARN) diabetic ketoacidosis (DKA) randomized trial [15] evaluated rapid or slow fluid administration to patients with diabetic ketoacidosis and found no difference in neurological outcomes. Small feasibility randomized trials have been designed in the adult setting to prospectively evaluate the clinical effects of positive FB, such as REVERSE AKI (evaluating restrictive fluid protocols vs. standard of care in AKI) that was able to show a more negative FB in the intervention group with a reduction of adverse events [16]. Similarly, the role of active deresuscitation after resuscitation-2 (RADAR-2) study confirmed the feasibility of forcing a negative FB with a restrictive strategy in patients with fluid accumulation [17^▪^]. Authors from low-income countries are similarly evaluating the translatability of these findings, because due to limited resource differences the effects of positive fluid balance may be more detrimental. This was demonstrated in the Fluid Expansion as Supportive Therapy (FEAST) trial that found higher mortality with more fluid boluses in febrile children compared to no fluid boluses [18]. This study, however, was limited by the absence of heart or kidney function monitoring in sick patients. There is currently an ongoing study on slow rehydration in gastroenteritis for severely malnourished children vs. standard of care with World Health Organization plans in sub-Saharan Africa [19].

The other potential underlying difference in contradictory results of studies on FB and outcomes relies on the assumption that total body FB is akin to intravascular volume status. There are benefits to using total body FB measurements: often more comparable across hospitals and different clinical settings, relative ease of obtaining, historical context, albeit with some limitations of the metric-based system that often relies on invasive Foley catheter to truly obtain accurate outputs. Emerging evidence in adult patients shows that intravascular volume status may be more important when assessing responsiveness to fluid addition or removal. To assess the intravascular FB, on the other hand, there is less consensus on the best ways to measure this. Common methods often combine multiple data points that include physical exam findings (e.g., heart rate, blood pressure, edema, crackles on lung auscultation), laboratory values (e.g., hemoconcentration trends such as hemoglobin and/or sodium, rises in lactate), and chest X-ray findings of pulmonary edema or B-lines on lung ultrasound. A recent observational analysis among 50 mechanically ventilated children showed that there was not a correlation between positive FB (either weight-based or metric-based) and central venous pressure measurements, suggesting that our current measurements for FB remain sub-optimal and therefore difficult to study [20^▪^].

Dynamic measurements have been evaluated and showed promise compared to static measurements for assessing responsiveness to fluid interventions [21]. In a large meta-analysis of 2260 adult hemodynamically unstable patients, Bentzer *et al.*[22] found that half of patients were not fluid responsive, and that the augmentation of cardiac response after passive leg raising was the best predictor with a positive likelihood ratio (LR) of 11 (95% CI 7.6–17.0). The next best indicators of fluid responsiveness were dynamic measurements (inferior vena cava distensibility/collapsibility had a positive LR of 5.3 (95% CI 1.1–27), and a central venous pressure measurement <8 had a positive LR of 2.6), while static physical examination findings were not predictive of fluid responsiveness. A recent randomized control trial in critically ill adults found that although a focused fluid assessment with integrated point of care ultrasound (POCUS) was associated with a shorter duration of vasopressor use, this was achieved at the cost of higher fluid balance compared to standard of care. However, there were no differences in either for 28-day mortality or ICU length of stay [23]. Unfortunately, so far, dynamic indexes of fluid responsiveness have shown to be suboptimal in the pediatric setting and novel techniques should be validated in this field [24].

## POSITIVE FLUID BALANCE IN SPECIFIC POPULATIONS IS AT HIGH RISK TO DEVELOP FLUID OVERLOAD

According to the described controversies regarding the issue of fluid overload, it can be stated that each critically ill child should be strictly monitored regarding cumulative FB. The exact threshold to distinguish positive FB from fluid overload is currently not known. Also, the same percentage of cumulative FB can have different effects on children with different clinical conditions or severity of disease. However, even if in clinical practice FB should be critically assessed in different patients, it must be highlighted that the threshold of 20% of cumulative positive fluid balance in critically ill children has been associated with a threefold higher risk of mortality in a large retrospective study on >1000 patients [25^▪▪^].

For sure, specific subpopulations show a particularly high sensitivity to excessive fluid administration. A recent meta-analysis on >3000 cardiac surgery children, demonstrated that fluid overload was linked to significantly higher risk of mortality, AKI, incidence of infection, prolonged cardiac intensive care unit stay and increased duration of mechanical ventilation and vasoactive drugs requirement [26^▪^]. These data should be interpreted with caution since a standard definition is lacking among different authors. The threshold of fluid overload ranged from a cumulative positive fluid balance of 2.7–20%, peaking from the first up to the fifth postoperative day. Interestingly, the analysis showed a linear correlation between fluid overload and outcomes (in particular, with mortality and AKI) [26^▪^]. It must be remarked that these studies were heterogenous and different authors observed the effect of positive FB in different groups of cardiac children (i.e., neonates, surgical patients, subjects undergoing extracorporeal treatments, etc.), exploring personalized FB thresholds. Hence, it is currently impossible to determine an absolute value of positive FB that should be considered as pathologic (i.e., fluid overload) in cardiac children. In other words, even if these patients are certainly at higher risk to develop a pathologic state of FB, several cofactors (i.e., age, cardiac anatomy, duration of cardiopulmonary bypass, preoperative hemodynamic instability, or renal function) should be taken into account when evaluating their fluid management.

Another important field regarding the management of FB is pediatric respiratory failure. Some evidence is emerging about the impact of fluid overload in pediatric acute respiratory distress syndrome (pARDS). Leow *et al.*[27^▪^] showed in 165 patients with moderate to severe pARDS that the combination of AKI and peak cumulative FB were associated with mortality and ventilation free days, respectively. Interestingly, this study found that the rate to peak fluid overload (i.e., the amount of daily positive FB increase) was about 4% in nonsurvivors and was associated with mortality after adjustment for confounders. The importance of the time dependent effects of FB was also showed by Black *et al.*[28^▪▪^], who concluded their retrospective analysis on >700 children with pARDS, stating that in multivariable analysis, a positive cumulative fluid balance on days 5 through 7 was associated with increased mortality and that higher cumulative fluid balance on days 4 to 7 was associated with lower probability of extubation.

A sub-type of children at higher risk of accumulating positive fluid balance is also composed by patients undergoing extracorporeal membrane oxygenation (ECMO), since significant amounts of fluids are prescribed to treat hypotension either due to hypovolemia or reduced vascular systemic resistances [29]. In these cases, fluids may also be prescribed because hypovolemic children undergoing extracorporeal life support undergo circuit chattering or because of bleeding. In these patients, positive FB becomes rapidly a cofactor of multiple organ dysfunction, mainly due to systemic congestion and/or suboptimal perfusion. Kidneys are typically involved in such complication secondary to fluid overload, and AKI incidence ranges from 30% to 80%, compared to critically ill children without ECMO (10–20%), further increasing the difficulty of FB management [29]. Gist and coworkers recently reported an analysis on more than 130 children undergoing ECMO indicated as cardiopulmonary resuscitation. They found that the presence of fluid overload at ECMO discontinuation (defined as a cumulative positive FB above 10%), but not AKI was associated with mortality, further reinforcing the concept that excess fluids may be harmful in high-risk children, regardless of clinical phenotype (i.e., AKI positive or AKI negative) [30]. Gorga *et al.*[31^▪^] also showed in a multicenter survey that fluid management represents the predominant indication for initiation of CRRT in ECMO children and that this indication has significantly increased in the last 20 years, showing that the attention of clinicians to this issue has greatly improved.

## MANAGEMENT OF POSITIVE FLUID BALANCE AND FLUID OVERLOAD

In a meta-analysis focusing on the association between fluid overload and outcomes in critically ill children, Alobaidi *et al.*[1] also examined this issue in a subgroup of studies regarding patients receiving CRRT. In these cases, when fluid overload was evaluated as a continuous exposure, survivors had lower percentage fluid overload compared with nonsurvivors. This association appears stronger than in patients with sepsis, acute lung injury or other general diagnoses. Although based, again, mainly on observational reports, this notion remarks the importance of an accurate and possibly proactive management of FB through accurate prescription of net ultrafiltration and constant control of FB on clinical charts, avoiding the occurrence of positive FB exceeding the threshold of 10–20% or treating it effectively. On this light, as a first prospective attempt to control positive FB and potentially impact patients’ outcomes, Nelson and coworkers conducted a project aiming, through educational initiatives and daily calculation of net percentage fluid overload on electronic charts, to start CRRT before the threshold of 20% positive fluid balance was reached [32^▪▪^]. These authors were able to show that they were able to meet this target in the majority of their patients and that this timing was reached in a 72% larger number of patients with respect to the era before the project was started. Decongestive treatments (both medical, through the liberal use of diuretic agents, and extracorporeal) have also been prospectively evaluated in the RADAR-2 trial, conducted in critically ill adult patients [17^▪^]. However, so far, unequivocal evidence of the beneficial clinical effects of timely and aggressive downloading of FB in the post resuscitation phase, in terms of mortality and ventilation duration or hospital stay, is not available, although these initial pivotal trials seem promising.

## CONCLUSION

Currently, no accepted definition of FB and fluid overload has been described, however it is clear that they are two clearly distinguished clinical conditions. Accurately monitoring of FB is reasonable in high-risk critically ill patients (i.e., post-cardiac surgery, those with respiratory failure, those undergoing extracorporeal circulation) since this positive FB can easily become fluid overload in these subpopulations. However, it is currently unclear if prevention of specific levels of positive FB through fluid restrictive regimes or timely decongestive therapies can improve patient outcomes and novel prospective evidence is currently ongoing.

## Acknowledgements

*None.*

### Financial support and sponsorship

*None.*

### Conflicts of interest

*There are no conflicts of interest.*

## REFERENCES AND RECOMMENDED READING

Papers of particular interest, published within the annual period of review, have been highlighted as:

▪ of special interest
▪▪ of outstanding interest
